# Adaptability in the aperiodic *Drosophila* populations and evolution of the life-history traits.

**DOI:** 10.17912/micropub.biology.001436

**Published:** 2025-03-23

**Authors:** Khushboo Sharma, Nalini Mishra, Mallikarjun N. Shakarad

**Affiliations:** 1 Department of Zoology, Banaras Hindu University, Varanasi, Uttar Pradesh, India; 2 Department of Zoology, University of Delhi, Delhi, India; 3 Regional Medical Research Centre, Gorakhpur, India

## Abstract

Exposure of diurnal animals to constant light for extended periods dampens the clock gene circadian rhythms, which in turn affect the life history traits. However, animals are expected to maintain some form of rhythm for the body to function effectively. In this study, we used three populations of *Drosophila melanogaster* that were maintained under constant light for 312 generations. We entrained three other populations (derived from those in constant light for 312 generations) to 12L:12D cycles. The adaptive evolution under aperiodic conditions has compromised on longevity and fecundity- two most important fitness traits for an iteroparous species.

**Figure 1. Effect of LD entrainment on the Eclosion pattern across generations and life-history traits.**
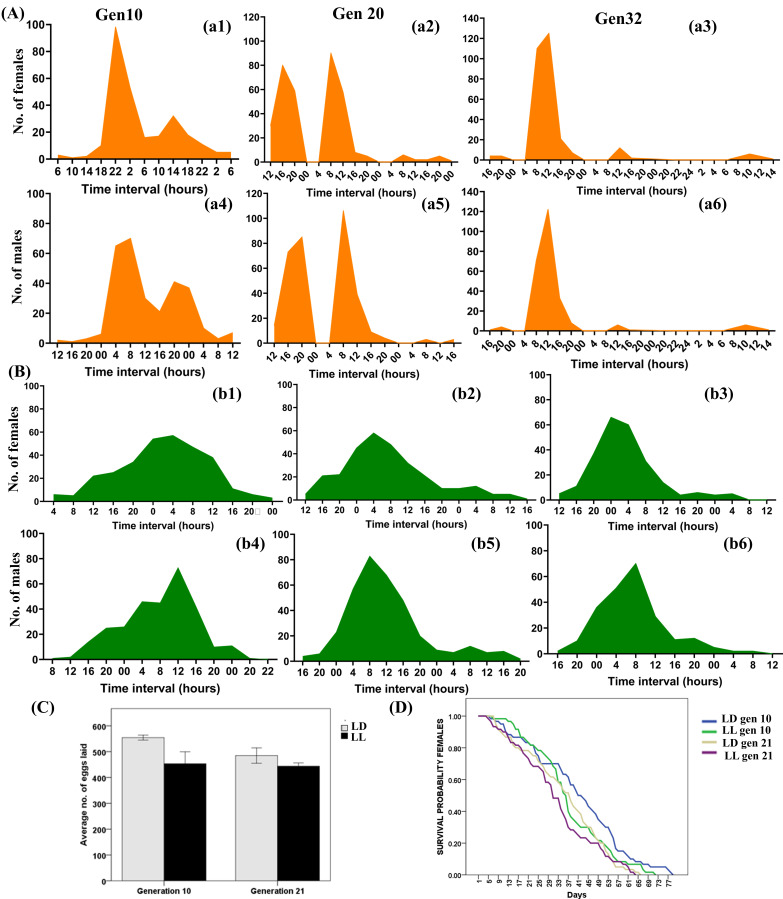
(A-B) Gender specific eclosion pattern across generations gen. 10 (a1, a4; b1, b4); gen. 20 (a2, a5; b2, b5); gen. 32 (a3, a6; b3, b6); whereas a1-a6 are LD (12h Light and 12h Dark) populations and b1-b6 are LL (24h Light and 0h Dark) populations; N= 750 per regime. (C) Average lifetime egg laying in LD and LL populations, (N= 60 pairs per regime) (D) Survival probability of females of LD and LL populations across generations. LD females have extended lifespan than LL population females (N= 60 per regime) whereas colours- blue and green codes for generation 10 and beige and purple for generation 21 of LD and LL, respectively.

## Description

An organism’s biological clock may have daily rhythmicity due to (i) entrainment i.e., synchronization to environmental cycle *viz* ., light and temperature or (ii) free running i.e., endogenous periodicity (Giebultowicz, 2018). This might help the organisms to have appropriate phase relationship of their behavior and physiology with the extrinsic cyclic environmental factors for its survival. The anticipated daily cycles due to light and dark allows synchronization of physiology and behavior of the organism with respect to external stimuli/ environment (Giebultowicz, 2018). The daily light-dark cycle is most consistent and reliable entraining cue for the daily clock of an organism. The circadian disruption caused by exposure to continuous light might have negative influence on the physiology and fitness of the organisms (Bedrosian and Nelson, 2017; Chalfant et al., 2020). The circadian rhythm dysfunction due to genetic mutations and or environmental changes negatively affects major life-history traits in *Drosophila melanogaster* (Allemand et al., 1973; Beaver et al., 2002, 2003; Kumar et al., 2007). Longevity and fecundity of flies is affected by eclosion time of the flies that itself is influenced by light-dark cycles as well as gender of the flies (Nikhil et al., 2016). The present study was designed to understand the effect of continuous light/ aperiodic condition, which act as a source of light pollution i.e., excess of artificial light on life history traits of *Drosophila melanogaster* across generations. We assessed whether or not the intrinsic biological rhythms are preserved after being in continuous light condition for 312 generations.

Here, we re-introduced light dark cycle i.e., the L:D entrainment to understand their impact on life-history traits across generations. We show that entrainment with 12L:12D cycle of *Drosophila* populations that were under aperiodic condition of 24L:0D condition for more than 312 generations, resulted in rhythmic eclosion. The eclosion pattern was unaltered post 10 generations of exposure to L:D entrainment. However, the eclosion rhythm became apparent post 20 generations of L:D entrainment. The entrained clock affects the fecundity and enhances the longevity, suggesting circadian rhythmicity positively affects physiology of flies.

Synchronous emergence of individuals of a given species ensures the availability of physiologically similar individuals in a population, thus maximizing the evolutionary fitness. In *Drosophila* , the “gated emergence” from pupa to adult is tightly regulated and precise (Kannan et al., 2012a) and is enhanced by both, precision and accuracy of circadian gating. A recent study reported that larval density dependent developmental manipulation does not affect the accuracy of eclosion in flies and that gating period restriction is responsible for the eclosion phase (Varma et al., 2019). We found that under continuous light conditions circadian gating is absent while entrainment with LD conditions lead to circadian gating and restoration of eclosion rhythm (Fig. 1) suggesting persistence of eclosion rhythms in the aperiodic conditions (Sheeba et al., 1999b). Arrhythmic eclosion pattern changed into a rhythmic pattern under LD condition. The average eclosion time of similar control populations (CP) maintained under constant light condition are 170 h and under L:D cycle is 211 h (Varma et al., 2019). However, in our study, we entrained the populations across generations under 12 h of Light and 12 h of Dark cycle and determined the eclosion pattern across generations. The average L1 to adult development time for males after 10, 20 and 30 generations under LD condition were 216 h, 198 h and 193 h respectively; and the corresponding values for females were 208 h, 188 h and 193 h. The corresponding development time under LL condition for the male flies were 211h, 210 h and 207 h; and for females they were 204 h, 208 h and 201 h. However, the differences are not statistically significant. Our results are in concordance with those of Varma et al. (2019), in that we also observed the differences in eclosion profile due to gating by circadian clock rather than differences in developmental rate.

Longevity and fecundity are two important life-history traits (Flat 2011). We examined effect of entrainment with 12L:12D cycle in populations that were under continuous light exposure. The homogeneity of variance was assessed using Bartlett test where variance was found to be equal. There was significant effect of light regime on life-time oviposition (F _1, 2_ = 36.614, p = 0.026, Fig. 2A). Females under 12L:12D condition laid on an average 554 and 484 eggs while females maintained under constant light condition laid an average of 453 and 443 eggs, in generation 10 and generation 20 respectively. However, there was no effect of generations on lifetime egg laying (F _1, 2_ = 3.123, p = 0.216). We compared the survival probabilities using Kaplan Meir analysis – a non-parametric test to assess age specific survival rates of female flies. There was significant effect of LD condition on survival probabilities of females. The median lifespan of LD females was 41 days and 36 days while median lifespan of LL females was 35 days and 30 days after 10 ^th^ and 20 ^th^ generation respectively. The flies maintained in 12L:12D conditions had significantly higher survival probabilities compared to those maintained under constant light conditions. Overall, constant light has affected the longevity of the flies which could be due to less robust clock gene rhythms.

In conclusion, our study suggests that there is intrinsic adaptive periodicity in population maintained under aperiodic condition. The entrainment with LD cycle was able to restore daily rhythms through alteration of clock gene expression that further affected two crucial fitness related traits- lifespan and fecundity.

## Methods

**Fly stock and maintenance**

The populations used in the present study were derived from Joshi Baseline (JB) populations that were on a 21-day discrete generation cycle (Sheeba et al., 1998) under standard laboratory conditions (SLC) of 25 ± 1 ˚C, 75 ± 5% relative humidity and 24L:0D cycle as mentioned in Joshi and Muller (Joshi and Mueller, 1996). The maintenance protocol is described in Sharma et al., 2020. Briefly, the eggs were collected from running cultures of JB _1-4_ populations at a density of 50-60 eggs per 6 mL standard media. Ten egg vials were set up per population. All emerging adults were transferred into a pre-labeled population cage 12 days post-egg collection and aged for additional 6 days with food change every alternate day. Embryos were collected for starting the next cycle 21 days post egg-collection of the previous cycle.

**Light Dark Cycle introduction**

Three replicate populations each of two kinds of populations- LL (24h Light and 0h Dark) and LD (12h Light and 12h Dark) were initiated from JB populations. They were maintained under SLC and 24L:0D cycle for LL and 12h Light and 12h Dark for LD populations. The assays were performed on the LL and LD populations after 10 generations of maintenance under the above-mentioned conditions. Forty egg vials each with 50-60 eggs per 6 mL food vial per population were incubated. The temperature and humidity conditions were identical to that of the parental populations, while the light conditions differed. The LL populations were maintained under constant light conditions while LD populations were maintained at 12h L:12h D conditions. The lights on and off time for LD population was set at 0700 hours and 1900 hours respectively. The breeding population sizes in all cases were 1600-1800 flies per replicate population. Embryos for all assays were obtained from flies generated by incubating 40 vials with exactly 50 eggs/ 6 mL SM vial for 12 full days in their respective culture chambers. All emerging adults (hence forth referred to as standardized flies) were transferred to pre-labeled population cages and provided with fresh food plate (Sharma and Shakarad, 2021). All assays were set up during the subjective light phase of the LD populations.

**Eclosion rhythm assay**

Exactly 50 eggs were counted on non-nutritive agar-agar (12.5%) with fine brush, under Zeiss Stemi DV4 stereo zoom microscope and transferred to 6 mL SM vials. Five vials per replicate for each of the three replicate populations were set for LL and LD regimes. Hence, the total sample size, N = 750 per regime. After red eye spot formation, vials were under vigil check every 6 h till tanning of wings; following which monitoring for eclosion were carried out at 4 h interval. During the subjective night of the LD populations the eclosion checks were carried out under red light. The emerging adults were collected into clean dry pre-labeled vials. Using the eclosion census data we ascertained the eclosion pattern of LL and LD populations. Eclosion assays were conducted after 10-11, 20-21 and 31-32 generations in the respective light regimes. The eclosion pattern is used as assay of choice to study biological rhythm pattern.

**Longevity & Lifetime fecundity assay**

The flies collected from eclosion rhythm assay were held in unisex-holding vials for longevity & lifetime fecundity assay. Twenty mixed sex pairs (1male and 1female) were formed per replicate population and transferred to 3 mL SM vials. Three replicate populations were set up per light regime. Total sample size, N = 60 per light regime. The files were flipped into fresh SM vials every 24 hours and eggs laid during the preceding 24 hours counted using Zeiss Stemi DV4 stereo zoom microscope. The assay was continued till all flies died. From the same setup we extracted longevity data. Since the fitness in a closed (single pair) insect system is mainly influenced by the female longevity as she stores the sperms in her spermatheca and uses them to fertilize the eggs before releasing thus ensuring the male partner’s fitness; we have presented only female longevity data.

The life history traits *viz* ., L1 to adult eclosion pattern, longevity and fecundity were assessed for generation 10-11 and generation 20-21, for LL and LD populations. Replicate population means were used in all statistical analyses.

**Statistical analyses:**

In all cases except survival probability function, factorial-ANOVA, was performed using replicate population means and graphs were prepared using SPSS v. 22. Since population means were used as units of analysis with selection, fly gender and generation number as fixed variables and replication as a random variable only fixed-factor effects and their interactions could be tested for significance (Sharma et al., 2020). The significance of adult survival probability curves was analyzed using Kaplan-Meier log-rank test (Sharma and Shakarad, 2021).

## Data Availability

Description: Data for Average Egg laying across generations (Sheet 1); Eclosion time across generations (Sheet 2); Kaplan Meir Analysis across generations (Sheet 3). Resource Type: Dataset. DOI: https://doi.org/10.22002/6r74d-dpv16
